# Ursodeoxycholic Acid Improves Mitochondrial Function and Redistributes Drp1 in Fibroblasts from Patients with Either Sporadic or Familial Alzheimer's Disease

**DOI:** 10.1016/j.jmb.2018.08.019

**Published:** 2018-10-19

**Authors:** Simon M. Bell, Katy Barnes, Hannah Clemmens, Aziza R. Al-Rafiah, Ebtisam A. Al-ofi, Vicki Leech, Oliver Bandmann, Pamela J. Shaw, Daniel J. Blackburn, Laura Ferraiuolo, Heather Mortiboys

**Affiliations:** Sheffield Institute for Translational Neuroscience (SITraN), University of Sheffield, 385a Glossop Road, Sheffield, S10 2HQ, UK

**Keywords:** Drp1, dynamin-related protein, PSEN1, presenilin 1, Mfn1, mitofusin 1, Mfn2, mitofusin 2, Opa1, optic atrophy 1, UDCA, presenilin, treatment, neurodegeneration, mitochondrial morphology

## Abstract

Alzheimer's disease (AD) is the leading cause of dementia worldwide. Mitochondrial abnormalities have been identified in many cell types in AD, with deficits preceding the development of the classical pathological aggregations. Ursodeoxycholic acid (UDCA), a treatment for primary biliary cirrhosis, improves mitochondrial function in fibroblasts derived from Parkinson's disease patients as well as several animal models of AD and Parkinson's disease. In this paper, we investigated both mitochondrial function and morphology in fibroblasts from patients with both sporadic and familial AD. We show that both sporadic AD (sAD) and PSEN1 fibroblasts share the same impairment of mitochondrial membrane potential and alterations in mitochondrial morphology. Mitochondrial respiration, however, was decreased in sAD fibroblasts and increased in PSEN1 fibroblasts. Morphological changes seen in AD fibroblasts include reduced mitochondrial number and increased mitochondrial clustering around the cell nucleus as well as an increased number of long mitochondria. We show here for the first time in AD patient tissue that treatment with UDCA increases mitochondrial membrane potential and respiration as well as reducing the amount of long mitochondria in AD fibroblasts. In addition, we show reductions in dynamin-related protein 1 (Drp1) level, particularly the amount localized to mitochondria in both sAD and familial patient fibroblasts. Drp1 protein amount and localization were increased after UDCA treatment. The restorative effects of UDCA are abolished when Drp1 is knocked down. This paper highlights the potential use of UDCA as a treatment for neurodegenerative disease.

## Highlights

• Sporadic and PSEN1 fibroblasts share a changed mitochondrial phenotype.
• This phenotype consists of reduced membrane potential and altered morphology.
• Ursodeoxycholic acid corrects mitochondrial morphology and membrane potential.
• Ursodeoxycholic acid effects act via action on Drp1 quantity and localization.
• This paper highlights the potential use of UDCA to treat Alzheimer's disease.

## Graphical Abstract

**Figure:**
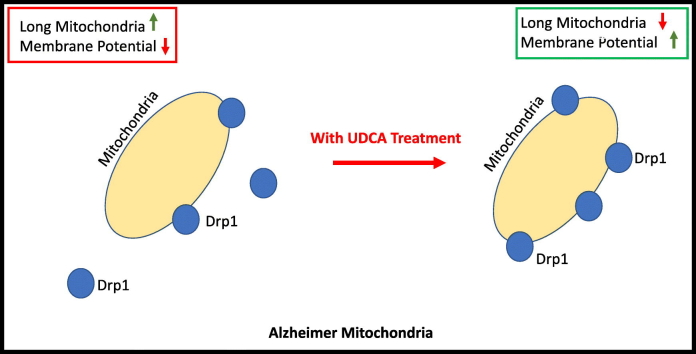

## Introduction

Alzheimer's disease (AD) is the leading cause of dementia worldwide and is characterized by the build-up of amyloid plaques and neurofibrillary tangles with a loss of neurons later in the disease course [1]. Mounting evidence indicates that amyloid plaques and neurofibrillary tangles do not correlate well with disease severity [2].

Mitochondrial dysfunction is a well-established mechanism in familial and sporadic forms of AD (sAD), with evidence from both post-mortem and peripheral patient tissue as well as animal models. Fluorodeoxyglucose positron emission tomography imaging in living patients has identified hypometabolism in parietal and temporal brain regions, even in early disease. Alterations in glucose metabolism and cellular respiration have also been found in AD patient fibroblasts [3], [4], [5], [6], [7]. Post-mortem data from AD patients show reduced activity of tricarboxylic acid enzymes and reduced complex IV activity, with complex IV activity decreasing during disease progression [8], [9]. Mitochondrial enzymatic failure, reduced glucose metabolism and increased reactive oxygen species production have all been shown to occur before amyloid pathology [10]. Expression of mitochondrial subunits from all respiratory chain complexes is reduced in the entorhinal cortex (which is an area of early pathological change in AD) of AD patients at post-mortem [11]. In addition, similar changes in expression of mitochondrial genes have been shown early in disease progression in whole blood samples of AD patients [12]. It is not only mitochondrial function that is altered in AD; of particular importance in neurons is mitochondrial dynamics. Mitochondria are in a constant state of flux undergoing fission and fusion events allowing them to adapt and meet local energy requirements. Evidence from both neurons and patient fibroblasts shows that mitochondria are more elongated and have altered distribution throughout the cell [6]. In particular mitochondria are localized around the perinuclear region in sAD fibroblasts suggesting a collapse of the mitochondrial network [13]. Mitochondrial dysfunction is a shared mechanism between sAD and familial forms of AD. Transgenic models of familial AD that incorporate amyloid precursor protein (APP) show impaired mitochondrial function and changes in mitochondrial morphology, specifically reduced mitochondrial membrane potential and tricarboxylic acid enzyme enzymes as well as reduced ATP levels [14], [15]. In addition, genetic risk factors for AD alter mitochondrial function. Possession of the *APOE*4 allele is the largest genetic risk factor for sAD, and possession of this allele is associated with reduced expression of respiratory chain complex proteins and activity of complex IV [16], [17]. Much work has been done trying to elucidate the mechanisms which cause AD with a view to finding therapeutic targets to slow or stop the progression of AD. To date, however, these interventions have not succeeded in modifying clinical outcome. The search for therapeutic targets has focused mostly around the amyloid cascade.

Mitochondrial abnormalities are also found in fibroblasts of patients with other neurodegenerative diseases. We and others have extensively characterized these changes in Parkinson's disease (PD) genetic subtypes [18], [19], [20], [21], [22], [23] and MND sporadic and genetic subtypes [24], [25]. We were the first to use the mitochondrial functional deficits as a primary screen in a drug screening cascade for PD [20]. We identified ursodeoxycholic acid (UDCA) in a drug screen of fibroblasts from *parkin* mutant PD patients, which we have subsequently validated in other forms of PD and other model systems [21]. UDCA is a promising compound as it is already in clinical use for the treatment of primary biliary cirrhosis.

We therefore hypothesized that mitochondrial abnormalities are detectable in fibroblasts from sAD and familial presenilin 1 (PSEN1) patients, and that these abnormalities could be improved with UDCA treatment. Here we describe our findings of mitochondrial membrane potential, mitochondrial morphology and localization, metabolic activity and mitochondrial fission/fusion machinery expression in sAD and PSEN1 fibroblasts. In addition, we describe a new mode of action of UDCA on mitochondrial respiration which is abolished when dynamin-related protein 1 (Drp1) is knocked down, indicating that Drp1 is involved in the recovery mechanism in AD.

## Results

### Mitochondrial function and morphology are altered in both sAD and PSEN1 patient fibroblasts

**Fig. 1:**
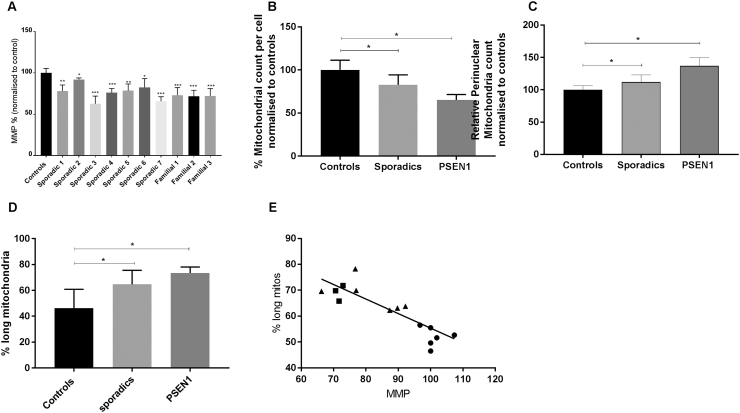
(A) Mitochondrial membrane potential in each of the seven sporadic and three familial AD lines. When compared to controls (*n* = 7), all sporadic lines and all familial lines showed a significant reduction in mitochondrial membrane potential (*p < 0.05, **p < 0.01, ***p < 0.005). (B) The total mitochondrial count was significantly reduced in both sporadic (*p < 0.05) and familial groups (*p < 0.05). (C) Perinuclear mitochondrial count was significantly increased in both sporadic (*p < 0.05) and familial (***p < 0.005) groups. (D) The percentage of cell area occupied by long mitochondria is increased in both sAD and PSEN1 patient fibroblasts (*p < 0.05). (E) Correlation of mitochondrial membrane potential and % long mitochondria shows negative correlation (*p* = 0.0002, *R*^2^ = 0.7). The circles represent controls; triangles, sAD; and squares, PSEN1. All measurements were carried out on three separate passages of each fibroblast line.

We initially investigated global mitochondrial function and morphology to address if there is a general mitochondrial phenotype present in AD. We assessed these mitochondrial parameters in two separate cohorts of fibroblasts from sAD patients, one collected locally (Sheffield cohort, *n* = 4) and one sourced from the Coriell cell repository (*n* = 3) in addition to a cohort of PSEN1 patient lines (*n* = 3 also sourced from Coriell cell repository and compared to controls (*n* = 4 from Sheffield and *n* = 3 from Coriell). We found reduced mitochondrial membrane potential in all sAD fibroblasts (controls 100 ± 5.3, sAD 83 ± 9; *p* < 0.05) and PSEN1 fibroblasts (controls 100 ± 5.3, PSEN1 71 ± 1.1; *p* < 0.01; Fig. 1A). Every AD fibroblast line had a significant reduction in mitochondrial membrane potential, ranging from a 35% to an 8% reduction.

In these same lines (both sAD cohorts and the PSEN1 fibroblasts), we also identified significant alterations in mitochondrial morphology and subcellular localization (Fig. 1B and C). We analyzed the mitochondria as three different entities: long mitochondria defined as mitochondria with form factor > 0.48, short mitochondria defined as mitochondria with form factor < 0.48 and all mitochondria. When considering total mitochondria, mitochondrial count per cell was reduced in the sporadic group (controls 100 ± 11.3, sAD 82.3 ± 11.5; *p* < 0.05; Fig. 1B) and in the PSEN1 group (controls 100 ± 11.3, PSEN 55.8 ± 18.3; *p* < 0.05; Fig. 1B). In both the sporadic and PSEN1 groups, the localization of mitochondria was focused more around the cell nucleus when compared to controls. Specifically, there was an increase in perinuclear mitochondria (controls 100 ± 6.2, sAD 112 ± 11.1, PSEN1 136.9 ± 12.9; *p* < 0.05 and *p* < 0.005 respectively; Fig. 1C). Both sAD and PSEN1 fibroblasts had a higher proportion of long mitochondria (% long mitochondria of total mitochondria controls 46 ± 14, sAD 65 ± 11, PSEN1 73 ± 11; Fig. 1D) indicating a more fused mitochondrial network. Conversely, the % of the cell area occupied by small mitochondria was decreased. The form factor of the overall mitochondria network was altered, which upon further investigation was due to the “small” mitochondrial components having higher form factor than in controls (data normalized to controls: controls 100% ± 3%, sAD 106% ± 2.5%, PSEN 111% ± 3%). We also found a correlation between the functional abnormalities and morphological changes; mitochondrial membrane potential and % long mitochondria showed a negative correlation (*p* = 0.0002, *R*^2^ = 0.7; Fig. 1E).

**Fig. 2:**
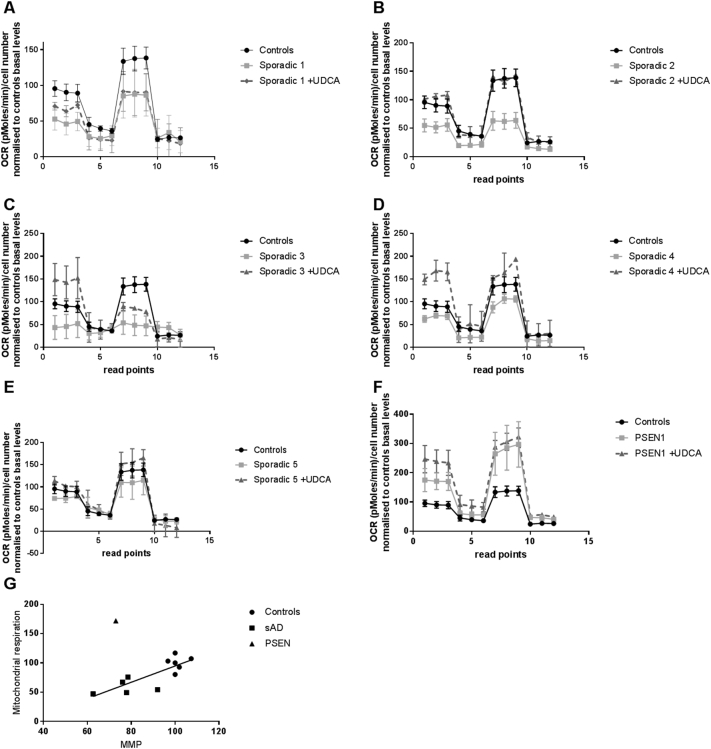
OCRs. Panels A–F show an OCR trace for controls (black circle solid line, *n* = 6), sporadic [gray squares and solid line, sAD1 (A), sAD2 (B), sAD3 (C), sAD4 (D), sAD5 (E)] and PSEN1 [gray squares and solid line, PSEN1 (F)] cell lines in the untreated condition. Sporadic patient fibroblasts have a reduction in OCR compared to controls at baseline and when measuring maximal capacity, but PSEN1 cell lines have an increase at both points. Panels A–F show the reduction seen in spare capacity in sporadic cell lines (*p < 0.05) and the increase seen in PSEN1 cell lines compared to controls (*p < 0.05). Fibroblasts after treatment with 100 nM UDCA for 24 h show increased mitochondrial respiration in both sAD (*p < 0.05) and PSEN1 (*p < 0.05) fibroblasts (A–F). Each measurement was repeated on three separate passages of each cell line. (G) Mitochondrial membrane potential shows correlation with spare respiratory capacity (*p* = 0.003, *R*^2^ = 0.65). The circles represent controls; squares, sAD; and triangles, PSEN1. The PSEN1 fibroblasts were excluded from linear regression calculations.

Next we investigated mitochondrial respiration in both sAD fibroblasts and PSEN1 mutant fibroblasts using the “mito stress test” on the Seahorse Analyser. This assay was undertaken on a limited number of sAD fibroblast lines (*n* = 5) and PSEN1 fibroblast lines (*n* = 2) due to poor growth of the other fibroblast lines. The Seahorse trace shows a reduction in oxygen consumption in each sAD fibroblast line measured compared to controls, whereas there is an increase in each PSEN1 patient fibroblast line (Fig. 2A–E). Specifically, we found a significant reduction in spare capacity in sAD fibroblasts of 34% when compared to controls (*p* < 0.05); this varied between a 15% reduction and a 35% reduction in the sAD fibroblasts; the variability is shown in Fig. 2A–E. The PSEN1 patient fibroblasts showed significant increases in mitochondrial respiration (increased by 52%, *p* < 0.01), ATP-coupled respiration (increased by 48%, *p* < 0.01) and spare capacity (increased 58%, *p* < 0.05; Fig. 2F). The reductions in spare mitochondrial capacity show correlation with mitochondrial membrane potential deficits (*p* = 0.003, *R*^2^ = 0.65; Fig. 2G); this is with the exclusion of the PSEN1 sample as this has reduced mitochondrial membrane potential, yet increased spare capacity. The Seahorse Analyser also simultaneously measures extracellular acidification rate (ECAR); we, however, did not find any significant alterations in basal or stimulated ECAR rates in the sAD or PSEN1 patient fibroblasts (data not shown).

### UDCA improves mitochondrial phenotype in sAD and PSEN1 fibroblasts

**Fig. 3:**
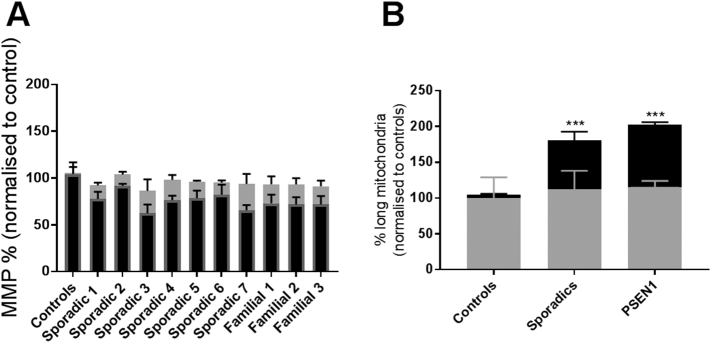
Recovery with UDCA treatment. (A) Fibroblasts are treated for 24 h with 100 nM UDCA. UDCA treatment, however, increases mitochondrial membrane potential. The increase from the untreated state for controls, sporadics and familial cell lines varies between 0 and 5% for controls, 12% and 28% for sAD and 19% and 21% for PSEN1 (*p < 0.05). (B) The increase in mitochondrial membrane potential when fibroblasts are treated with 100 nM of UDCA. The relative increase from the untreated state for controls, sporadics and familial cell lines is 0%, 11% and 25%, respectively (*p < 0.05). Each measurement was repeated on three separate passages of each cell line.

After identifying mitochondrial abnormalities in both sAD and PSEN1 patient fibroblasts, we next investigated if these mitochondrial parameters could be improved by treatment with UDCA. Treatment with 100 nM UDCA increased mitochondrial respiration and ATP-coupled respiration in both sAD and PSEN1 patient fibroblasts by 32% and 51%, respectively (Fig. 2F, *p < 0.05). There was no effect of UDCA on maximal capacity or uncoupled respiration. Treatment with UDCA also increased MMP (controls 2.1 ± 1.6, sAD 11.2 ± 2, PSEN1 24.7 ± 1.5; *p* < 0.05; Fig. 3A). This increase varied across AD fibroblast lines with an increase in mitochondrial membrane potential of between 12% and 28% (Fig. 3A). Furthermore, although the total mitochondrial morphology parameters were not significantly altered by UDCA treatment, the % long mitochondria was significantly reduced (controls + 5 ± 2.8, sAD − 24.5 ± 10.7, PSEN1 − 40.2 ± 2.1; *p* < 0.05; Fig. 3B).

### Mitochondrial morphology proteins are changed in AD patient fibroblasts; UDCA modulates mitochondrial fission proteins

**Fig. 4:**
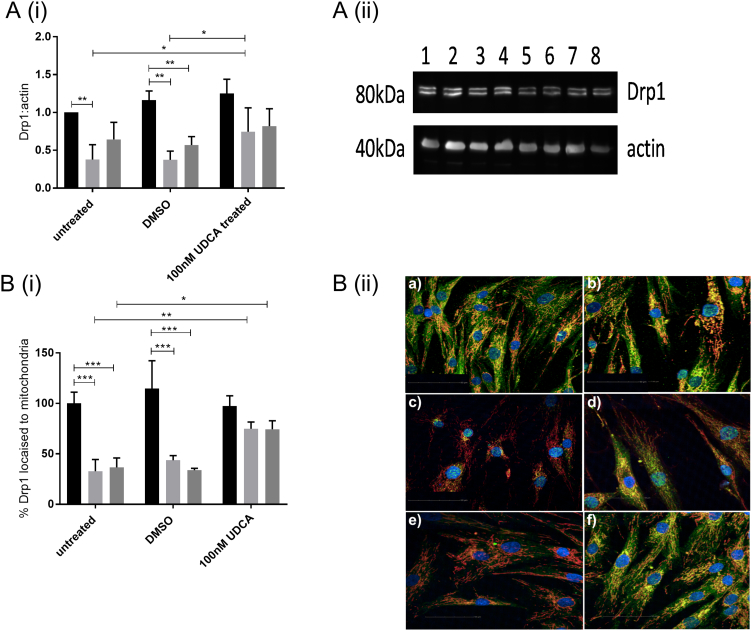
Drp1 protein expression. (A) Total Drp1 protein expression levels are reduced in both sAD (light gray bars) and PSEN1 (dark gray bars) fibroblasts (**p < 0.01). After treatment with 100 nM UDCA, total Drp1 levels are increased in the sAD fibroblasts (*p < 0.05). A(ii) Representative western blot showing control untreated (lane 1), control DMSO treated (lane 2), control 100 nM UDCA (lane 3), control 10 μM UDCA (lane 4), sAD untreated (lane 5), sAD DMSO treated (lane 6), sAD 100 nM UDCA treated (lane 7) and sAD 10 μM UDCA treated (lane 8) for Drp1 and loading control actin. (B) Drp1 cellular localization is altered in sAD and PSEN1 fibroblasts. Significantly less Drp1 colocalizes with the mitochondrial marker (TOMM20) in the sAD (light gray bars) and PSEN1 (dark gray bars) fibroblasts (***p < 0.005); however, after treatment with 100 nM UDCA for 24 h, the amount of Drp1 which colocalizes with the mitochondria is increased (*p < 0.05; **p < 0.01). B(ii) Representative images of control fibroblasts vehicle treated (a) and treated with UDCA (b) and sAD fibroblasts vehicle treated (c) and treated with UDCA (d) and PSEN1 vehicle treated (e) and treated with UDCA (f). Blue staining is Hoechst for the nucleus, green staining is Drp1 and red staining is TOMM20 for the mitochondria. Each measurement was repeated on three separate passages of each cell line; for immunocytochemistry measurements, at least 150 cells were imaged per fibroblast line per experiment.

**Supplementary Fig. 1:**
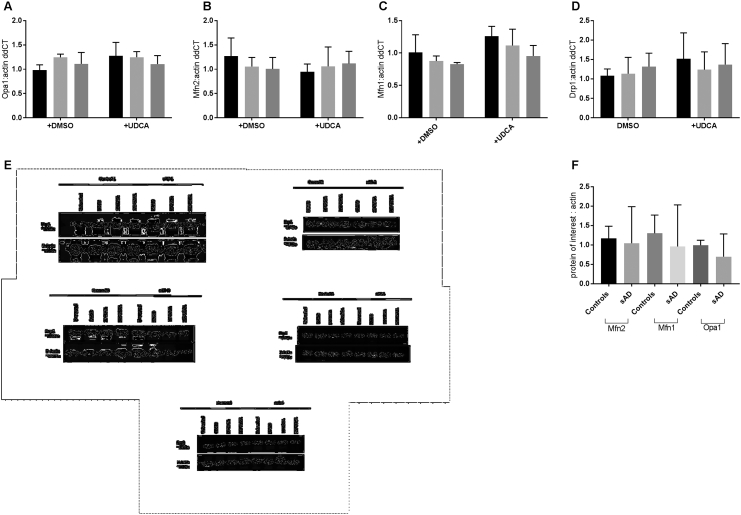
A-D show qPCR measurements of mRNA expression of Opa1 (A), Mfn1 (B), Mfn2 (C) and Drp1 (D). Black bars show controls, light grey bars show sporadic AD patient fibroblasts and dark grey bars show PSEN1 patient fibroblasts. Samples from each fibroblast line were run in triplicate and data presented is all controls, sAD and PSEN1 fibroblasts grouped. No significant differences are present in mRNA expression of any transcripts measured. Panels E and F show western blot data for Drp1, Opa1, Mfn1 and Mfn2. E shows individual western blot from each sAD fibroblast line sAD1-5. Showing a reduction in Drp1 protein levels in all sAD patient fibroblasts measured and an increase after UDCA treatment. Protein levels of Opa1, Mfn1 and Mfn2 were not changed in the sAD fibroblasts as quantified in F.

We investigated both mRNA and protein expression of the mitochondrial fission and fusion modulators Drp1, mitofusin 1 (Mfn1), Mfn2 and optic atrophy 1 (Opa1). We found no significant alterations in mRNA expression of any mitochondrial fission/fusion modulators in the sAD or PSEN1 patient fibroblasts (Supplementary Fig. 1A–D). Furthermore, we assessed the protein expression of the same mitochondrial modulators and found a reduction in the total cellular protein expression of Drp1 in both sAD and PSEN1 cohorts (controls 100 ± 12.2, sAD 37.8 ± 19.7, PSEN1 64.5 ± 22.5; *p* < 0.05; Fig. 4A). Example Western blots from each fibroblast line are given in Fig. 4A(ii) and Supplementary Fig. 1E. Total levels of Drp1 expression were increased after treatment with UDCA in sAD [sAD dimethyl sulfoxide (DMSO) treated 37.4 ± 11.5, sAD UDCA treated 74.5 ± 31.6; *p* < 0.05; Fig. 4A]. Levels of the other mitochondrial modulators were unaltered at protein level (Supplementary Fig. 1G). We next assessed the subcellular distribution of Drp1 using immunofluorescence staining. Drp1 is normally cytosolic and is recruited to the outer mitochondrial membrane at points of fission by receptors on the outer mitochondrial membrane such as mitochondrial fission 1 protein (Fis1), MFF, Mid49 and Mid51. Drp1 then acts in a pincer-like fashion to surround the outer mitochondrial membrane and pinch it together forming two daughter mitochondria. The quantification of our Drp1 staining showed that the AD patient fibroblasts (both sAD and PSEN1) have less Drp1 specifically at their mitochondria (controls 100 ± 11.13, sAD 32.77 ± 11.63, PSEN1 36.58 ± 9.35; *p* < 0.01; Fig. 4B). This indicates that Drp1 is not being recruited successfully to mitochondria. UDCA treatment increased the amount of Drp1 localized at the mitochondria membrane in both the sAD and PSEN1 patient fibroblasts (sAD DMSO treated 43.66 ± 4.62, sAD UDCA treated 74.88 ± 6.67, PSEN1 DMSO treated 33.86 ± 1.83, PSEN1 UDCA treated 74.5 ± 8.2; *p* < 0.05; Fig. 4B).

### Drp1 knockdown abolishes UDCA protective effect in sAD and PSEN1 fibroblasts

**Fig. 5:**
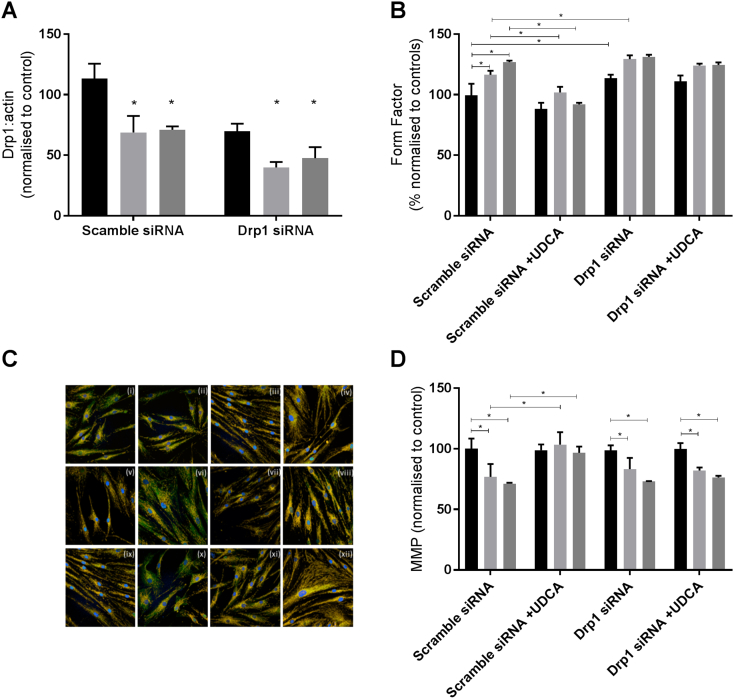
Drp1 knockdown data. (A) Drp1 protein expression knockdown of 48% from scramble siRNA levels (*p < 0.05). (B) Mitochondrial form factor is increased in sAD and PSEN1 fibroblasts in the scramble siRNA condition, which is reduced again after UDCA treatment. Drp1 knockdown increases form factor in all fibroblasts. UDCA does not have an effect on mitochondrial form factor in the Drp1 knockdown condition. (C) Image showing Drp1 staining in green, mitochondria in red and nuclei in blue. A representative image is shown for each condition. Control scramble siRNA (i), control scramble siRNA + UDCA (ii), control Drp1 siRNA (iii), control Drp1 siRNA + UDCA (iv); sAD scramble siRNA (v), sAD scramble siRNA + UDCA (vi), sAD Drp1 siRNA (vii), sAD Drp1 siRNA + UDCA (viii); PSEN1 scramble siRNA (ix), PSEN1 scramble siRNA + UDCA (x), PSEN1 Drp1 siRNA (xi), PSEN1 Drp1 siRNA + UDCA (xii). (D) Mitochondrial membrane potential is reduced in sAD and PSEN1 fibroblasts in the scramble siRNA condition, which is increased with UDCA treatment. In the Drp1 siRNA condition, there is no further decrease in mitochondrial membrane potential; however, UDCA treatment does not increase mitochondrial membrane potential.

To elucidate if the protective effect of UDCA was mediated by Drp1, we undertook knockdown experiments of Drp1 in control and AD patient fibroblasts. We achieved a knockdown of Drp1 protein levels at 48 h post-transfection of 40% compared to scramble siRNA negative control for each fibroblast line (Fig. 5A). This meant that the Drp1 level in the AD fibroblasts after knockdown was 25% of control levels as Drp1 levels are already lower in AD fibroblasts. Drp1 knockdown in control fibroblasts did not result in reductions in mitochondrial membrane potential; however, mitochondrial morphology was altered. Mitochondria were more elongated [form factor (normalized to controls as %) controls 100 ± 5, controls with Drp1 k/d 113 ± 3.3; *p* < 0.05, Fig. 5B] with an increased amount of the cell area occupied by long mitochondria (controls scramble 46 ± 2.3, controls Drp1 k/d 59 ± 3.4; *p* < 0.05). Similar changes were observed in AD fibroblasts after Drp1 knockdown with no effect on mitochondrial membrane potential but increased further % of cell area occupied by long mitochondria (sAD scramble 65 ± 3.4, sAD Drp1 k/d 74.4 ± 4.5; PSEN1 scramble 72.7 ± 5.3, PSEN1 Drp1 k/d 81.2 ± 5.2). However, the increase in mitochondrial membrane potential observed in AD fibroblasts after UDCA treatment was not seen in the Drp1 knockdown condition (Fig. 5D). Similarly, the “corrections” in mitochondrial morphology seen after UDCA treatment were not seen in the Drp1 knockdown condition (Fig. 5B). We investigated Drp1 cellular localization under conditions of Drp1 knockdown. We observed, as expected, less Drp1 colocalizing with mitochondria in all cells with Drp1 knockdown; UDCA treatment was not able to change this (Fig. 5C).

## Discussion

Here we show that detectable alterations in both mitochondrial function and morphology are found in peripheral fibroblasts from two separate cohorts of sAD patients and a group of PSEN1 familial AD patients. Mitochondrial membrane potential is reduced in all seven sAD fibroblast lines tested and the three PSEN1 fibroblast lines. It is noteworthy that we did not stress the cells at any point in order to reveal these mitochondrial abnormalities; they were identifiable under “normal glucose” culture conditions. Mitochondrial membrane potential was more variable in the AD patient fibroblasts than the control fibroblasts. We postulate that this is a feature of investigating sAD patients; the underlying cause of sporadic disease and disease stage at which each biopsy was collected is not known for every patient. These factors are likely to increase variability in these mitochondrial measures more than in controls without a neurodegenerative condition.

Abnormalities in the mitochondria of patients have been shown before [4], [6], [26], and it has also been shown that the amyloid protein itself can affect the function of mitochondria [27]. This is one of the first studies to directly compare sAD and familial AD patient derived fibroblasts at the same time. Although both sAD and PSEN1 patient fibroblasts have mitochondrial abnormalities, the level of defect varies between sAD and PSEN1 fibroblasts. Particularly striking is the increase in mitochondrial respiration in the PSEN1 mutant fibroblasts, whereas the spare respiratory capacity was reduced in the sAD fibroblast cohort. These biochemical data are difficult to interpret; the opposite effects found when investigating respiration and mitochondrial membrane potential in PSEN1 patient fibroblasts may suggest that the mitochondria are uncoupled. Some evidence for this can be gained from the Seahorses traces as the coupling efficiency is reduced in PSEN1 fibroblasts even if respiration is increased. Alternatively, as fibroblasts do not rely heavily on oxidative phosphorylation in order to maintain energy state, particularly in glucose media, this could suggest that PSEN1 mutants rely heavily on alternative energy pathways. This would best be investigated in additional cell types which rely to varying degrees on glycolysis *versus* oxidative phosphorylation. The recent study by Sonntag *et al*. [7] investigated mitochondrial respiration in a cohort of late-onset AD patient fibroblasts and found the mitochondrial respiration rates to be higher than controls, similar to the PSEN1 fibroblast lines we tested here. The apparent discrepancy between our sAD and the study by Sonntag *et al*. could in part be due to differences in the age of the patients, the cell culture conditions and passage of the cells at measurement. The sAD patients included in this study, particularly those from the Sheffield cohort, were young due to the patients being recruited at the Sheffield young-onset clinic, whereas those sAD patients biopsied in the study by Sonntag *et al*. were late-onset sAD patients. The age of the donors is also likely to explain the differences seen between our sAD cohort and that of Sonntag *et al*. with regard to cellular ATP levels. The global reductions in MMP we describe here largely concur with the previously published literature from AD patient fibroblasts (both familial and sporadic) [4], [6], [26], [28], [29]. The literature is clear that mitochondrial abnormalities are present and detectable in peripheral fibroblasts from AD patients.

The reasons for these abnormalities, however, are still being investigated. We show alterations in the protein expression and subcellular localization of Drp1. Drp1 levels have been implicated as a cause of mitochondrial abnormalities in sAD before, with reductions in Drp1 protein levels found in sAD fibroblasts [13], [29], which could be rescued by Drp1 over expression [13]. PSEN1 fibroblasts appear to be less affected than sAD fibroblasts. PSEN1 has been shown to increase activity at the mitochondrial associated membrane [30]; therefore, it may be having a direct role in controlling mitochondrial morphology, which is not present in sAD patients. The sAD cohort used in our study may have mitochondrial impairment as a more central pathological mechanism, and therefore, abnormalities are more pronounced when measured in particular in young onset sAD patients, as these patients mitochondria seem to have aged more rapidly than that of late-onset sAD patients and controls. The recycling of dysfunctional mitochondria is a process that Drp1 is also intimately involved with and has been shown by others to be defective in at least PSEN1 patient fibroblasts [29]. We did not investigate mitophagy in this report; however, this is a potential mechanism that could be investigated further.

Previous work by Manczak and colleagues [31] has shown an interaction between Drp1 and the amyloid protein in the brain of an APP mouse model. In this paper, partial reduction of Drp1 protein expression reduces the production of amyloid-beta and improves the mitochondrial dysfunction seen in an APP mouse model of AD. This same group has also shown that a reduction in Drp1 leads to a reduction in phosphorylated tau in the mouse brain [32]. It is plausible that the mis-localization of Drp1 that we have shown in our study is exacerbated in the brain by interaction with amyloid-beta as described in the above mouse models of AD. Our work further highlights the need to investigate Drp1 manipulation in other cell types such as neurons.

UDCA has been used in the treatment of primary biliary sclerosis for over 30 years. It has a limited side effect profile and is a relatively safe drug [33]. Furthermore, UDCA has been tested in several cell and animal models of AD and showed a putative protective effect [34], [35], [36], [37]. Here we show that UDCA restores mitochondrial membrane potential in both sAD and PSEN1 mutant fibroblasts; it, however, has no significant effect on mitochondrial morphology. However, we did find significant changes in the amount of Drp1 localizing to the mitochondria which increased in both sAD and PSEN1 fibroblasts, close to control levels. To verify if UDCA is acting via a pathway involving Drp1 in AD fibroblasts, we examined the protective effect of UDCA under a Drp1 knockdown condition; we found that knockdown of Drp1 levels abolished the protective effect of UDCA on mitochondrial membrane potential and mitochondrial morphology. This knockdown was transient as it was employed in the primary fibroblasts as a further validation sAD fibroblasts could be immortalized with subsequent Drp1 knockdown; further validation using single siRNA's would also strengthen this mechanistic link. As Drp1 knockdown in control cells did not result in significant reduction of mitochondrial membrane potential, it is plausible that UDCA modulation of Drp1 is part of a wider pathway altered by UDCA treatment without UDCA interacting directly with Drp1 itself. Drp1 is known to undergo a complex array of post-translational modifications including nitrosylation, ubiquitinylation, sumoylation and phosphorylation. In particular several phosphorylation sites have been identified; some promote mitochondrial fragmentation (via cyclin B) and others promote elongation via phosphorylation by protein kinase A. This phosphorylation site has been shown to inhibit disassembly of the Drp1 catalytic cycle, therefore accumulating large Drp1 oligomers on the outer mitochondrial membrane [38]. In this study, we did not investigate any of the post-translational modifications of Drp1 or any of the other known pathways that can affect Drp1 localization or phosphorylation state (including AMPK, protein kinase A and ERK); therefore, this should be investigated further to fully elucidate the mechanism of action of UDCA in AD. Furthermore, Drp1 has been implicated in the ER–mitochondrial contact sites (or mitochondrial associated membranes) where PSEN1 is known to increase activity in AD. The investigation of ER–mitochondrial contact sites is beyond the scope of this study; however, it would be important to investigate this pathway to assess the target by which UDCA is acting. Identifying the exact subcellular target by which UDCA is acting in AD is important for future therapeutic applications of UDCA and other potential neuroprotective compounds. Altering mitochondrial fission modulators has not been proposed before as a mechanism for UDCA and therefore warrants further investigation in other patient derived models and other animal models that have previously showed protection of UDCA.

In conclusion, our study has shown for the first time that reduced mitochondrial membrane potential in AD patient fibroblasts can be corrected with the treatment of UDCA via a pathway, which includes Drp1. Our study has identified a potential novel pathway by which UDCA has mitochondrial protective effects and further builds the case for the use of UDCA as a potential neuroprotective therapy for neurodegenerative disease.

## Methods and Materials

### Patient details

Fibroblasts from patients with sporadic and PSEN1 mutations were obtained from the NIGMS Human Genetic Cell Repository at the Coriell Institute for Medical Research: GM08243, GM07375, and GM07376 (all male, mean age 67.6 years, SD 6.65) and ND41001, ND34733, and ND34730 (2 male, 1 female, mean age 48.33 years, SD 11.06). These fibroblasts were compared with control fibroblasts (mean age 59.3 years, SD 6.35) from the same repository [GM07924, GM02189, GM23967 (all male)].

A second cohort of sporadic patient fibroblasts (2 male, 2 female, mean age 59.25 years, SD 5.37) and two aged matched controls (3 male, 1 female, mean age 60.66 years, SD 2.08) were also sampled from a local population of patients involved in the MODEL-AD research study (Research and Ethics Committee number: 16/YH/0155). The McKhannn *et al*. [39] criteria were used to diagnose AD, and participants took part in the European wide Virtual Physiological Human: Dementia Research Enabled by IT (VPH-DARE@IT).

### Cell culture

Primary fibroblast cells were cultured continuously using methods performed essentially as described by Mortiboys *et al* [24]. Cells were grown in EMEM (4500 g/L glucose), supplemented with 10% FBS, 100 UI/ml penicillin, 100 μg/ml streptomycin, 50 μg/ml uridine and 1 mM sodium pyruvate, 0.1 mM amino acids, and 0.1 × MEM vitamins. For mitochondrial membrane potential, morphology cells were plated at a density of 2500 cells per well in a black 96-well plate. UDCA (Sigma-Aldrich Ltd) was added to culture media (10 mM and 100 nM) 24 h prior to assay. DMSO was used as vehicle control.

### Mitochondrial morphology

Mitochondrial membrane potential and mitochondrial morphological parameters were measured using tetramethlyrhodamine staining of live fibroblasts. Briefly, 24 h after drug treatment, cells were incubated with 80 nM tetramethlyrhodamine and 10 μM Hoescht in phenol red-free media for 1 h. Cells were washed and imaged using the InCell Analyzer 2000 high-content imager (GE Healthcare). Raw images were processed and parameters obtained using a custom protocol in InCell Developer software (GE Healthcare) allowing for segmentation of mitochondria, nuclei and cell boundaries.

### Western blots

Cell pellets from patient and control fibroblasts were lysed using RIPA buffer and Protein Inhibitor Cocktail on ice, and protein levels were measured using a Bradford Assay. Twenty micrograms of protein was run on a 12% SDS-PAGE gel and transferred to a polyvinylidene fluoride membrane. Membranes were incubated overnight at 4 °C, with mouse anti-Drp1 (Abcam; 1:1000), mouse anti-OPA1 (BD Biosciences; 1:1000), mouse anti-Mfn1 (Abcam; 1:1000) or rabbit anti-Mfn2 (St Johns Laboratory; 1:500). Membranes were also probed for β actin as a loading control (St Johns Laboratory; 1:1000). Membranes were then incubated with goat anti-mouse HRP (Abcam; 1:10,000) or goat anti-rabbit HRP (Dako; 1:5000), as appropriate. Membranes were imaged using the G box chemi system using GeneSnap software (Syngene). Densitometry was analyzed using GeneTools software (Syngene).

### RNA extraction and qPCR

Fibroblasts were treated with UDCA 10uM and 100 nM 24 h prior to harvesting. Approximately 750,000 cells where harvested for each RNA extraction. RNA extraction was performed using an RNAeasy Plus Mini Kit (Qiagen) and by following Quick-start supplied protocol. RNA was converted into complimentary DNA using the QuantiTect Reverse Transcription Kit (Qiagen), standard Quick-Start supplied protocols were followed. qPCR was performed on a Stratgene PCR machine. Samples were loaded at 12.5 ng/μl per well. Sybr Green was used as the detection dye for the chain reaction, and a standard two-step program with 40 amplification cycles was used during the PCR. Primer sequences were as follows: Drp1: forward ATTATGCCAGCCAGTCCACAA, reverse CGCTGTTCCCGAGCAGATA; Mfn1: forward CACTCCAGCAACGCCAGATA, reverse CGGACGCCAATCCTGTTACT; Mfn2: forward GTCTGACCTGGACCACCAAG, reverse TGCAGTTGGAGCCAGTGTAG; and Opa1: forward AGCCAGTCCAAGCAGGATTC, reverse TGCTTTCAGAGCTGTTCCCT.

### Metabolic flux assay

Oxygen consumption rate (OCR) and ECAR were measured using a 24-well Agilent seahorse XF analyzer machine (Agilent). Human fibroblasts where plated at a density of 60,000 cells per well. Cells where treated with UDCA 100 nM for 24 h prior to measurement. Three measurements of OCR and ECAR were taken in each state: basal state, after the addition of oligomycin (0.5 μM), FCCP (0.5 μM) and rotenone (1 μM). A cell count was then done on a fixed assay plate using a Hoechst dye (1 μM). Data presented in this paper are normalized to cell number.

### Immunocytochemistry

Cells were fixed in 4% paraformaldehyde and then permeabilized with 0.1% Triton. After blocking, cells were incubated with anti-mouse Drp1 (BD Biosciences; 1:1000) and anti-rabbit TOM20 (Santa Cruz Biotechnology; 1:1000). Cells were then incubated with Alexa Fluor anti-rabbit 568 and Alexa Fluor anti-mouse 488 and 1 μM Hoechst. Cells were imaged using the Opera Phenix high-content imager (PerkinElmer). z Stacks were collected and analyzed using Harmony software (PerkinElmer).

### Drp 1 knockdown experiments

Cells were plated as above for the mitochondrial membrane potential and morphology assays. Cells were treated with either 100 nM Drp1 siRNA SMART pool Accell probes (Horizon Discovery) or 100 nM scramble siRNA-negative Accell probes in Accell delivery media. After 24 h, 100 nM UDCA was added to the treatment conditions, and 24 h later, the assays carried out.

### Statistical tests

Data are presented as normalized mean ± SD unless otherwise stated. For UDCA-treated parameters (ATP and MMP), data are presented as percentage increase from untreated basal levels. Data were analyzed using GraphPad Prism Software (V7.02): one-way ANOVA with Tukey's multiple comparisons posttest or, for UDCA-treated data, two-way ANOVA with Tukey's multiple comparisons posttest. In addition, *t* test was used to compare each individual AD fibroblast line to the control group for MMP.

The following are the supplementary data related to this article.
